# Transfer of Second Toe for the Treatment of Traumatic Thumb Amputation in a Four-year-old Child: A Case Report

**DOI:** 10.1055/s-0042-1744518

**Published:** 2022-05-31

**Authors:** Hugo Alberto Nakamoto, Reinaldo Borges Gonçalves, João Carlos Nakamoto, Fernanda do Carmo Iwase, Mariana Miranda Nicolosi Pessa, Marcelo Rosa de Rezende

**Affiliations:** 1 Grupo de Cirurgia da Mão e Microcirurgia, Instituto de Ortopedia e Traumatologia, Hospital das Clínicas (HC), Faculdade de Medicina (FM), Universidade de São Paulo (USP), São Paulo, SP, Brasil

**Keywords:** amputation, traumatic/surgery, child, thumb, toes/transplantation

## Abstract

Microsurgical toe transfer for thumb reconstruction is a challenging procedure, considering the technical skills necessary to perform it, as well as the difficult postoperative evaluation of esthetical and functional aspects. The present is the report of the case of a 3-year-old child who suffered a traumatic thumb amputation. Thumb reconstruction was performed through microsurgical toe transfer months after replantation failure. Subjective and objective outcome assessments were performed five years after the procedure. The functional outcome was evaluated through the Childhood Health Assessment Questionnaire (CHAQ) and the Jebsen-Taylor Hand Function Test (JTHFT). The Jamar dynamometer (Sammons Preston, Bolingbrook, IL, US) and the Jamar Pinch Gauge (Sammons Preston) devices were used to assess the handgrip and pinch strength respectively. The Semmes-Weinstein monofilament and two-point discrimination tests were performed. The patient presented an excellent functional outcome, partial recovery of strength, complete sensory recovery, and minimal donor site morbidity. A radiographic evaluation was also performed, and it demonstrated the preservation of the epiphyseal plate and the growth potential of the transplanted toe. In all aspects, the data observed reinforce the benefits and indications of toe transfer for thumb reconstruction in children.

## Introduction

Traumatic amputations of the upper limb are responsible for significant economic and social impacts. There are few epidemiological studies on finger amputations, but it is known that they are more frequent in young male adults. 1

Traumatic thumb amputation is rare and dramatic, and replantation should always be attempted. But, in case of failure, the procedure for thumb reconstruction, which includes distraction of the remaining part, pollicization, osteoplastic reconstruction, and toe transfer, should be considered. 2

Among all available procedures, toe transfer is the most challenging. However, it has great potential to provide a good final outcome. 3

Considering the many aspects involved in toe transfer, especially in children, we present a case highlighting the long clinical follow-up and the preservation of the growth potential.

## Case Report

The patient's parents signed the free and informed consent form. The present study was approved by the Ethics Committee of the hospital under number 4.075.812.

In 2013, a 3-year-old male child suffered a traumatic amputation of his right thumb by a circular saw. Replantation was performed; however, due to failure, the thumb had to be removed on the following week. In 2014, transfer of the second left toe to the right thumb was performed when the child was 4 years old.

Two surgical teams performed the procedure. During the dissection of the second toe, we identified the first dorsal intermetatarsal artery, which was the dominant artery, as well as two veins, two digital nerves, and the flexor and extensor tendons. Simultaneously, the hand dissection included identifying the radial artery, the recipient veins, the proper digital nerves, and the extensor and flexor pollicis longus tendons.

**Fig. 1:**
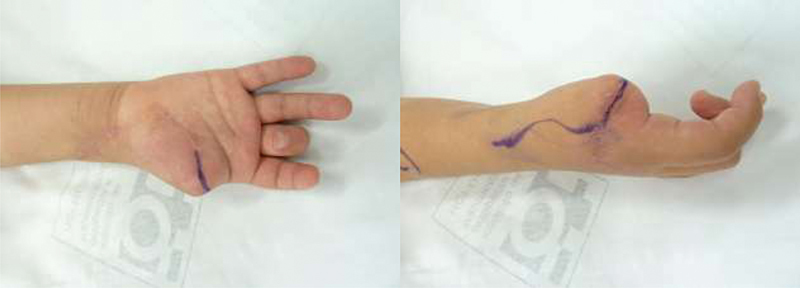
Planning of the hand incision.

**Fig. 2:**
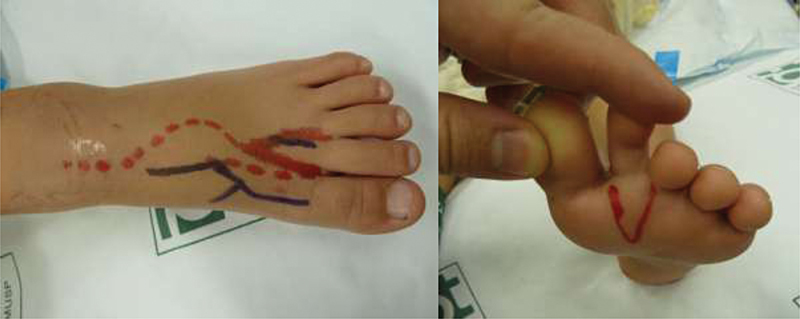
Planning of the foot incision.

**Fig. 3:**
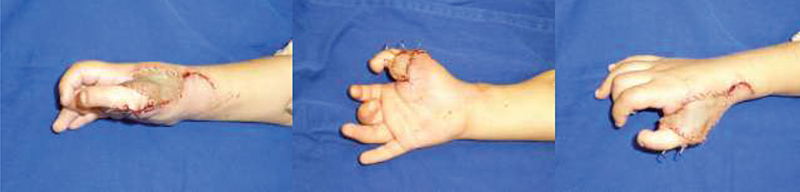
Immediate postoperative outcome.

**Fig. 4:**
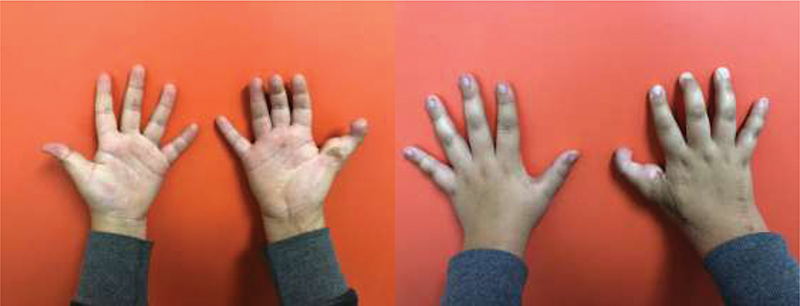
Esthetic and functional late postoperative outcome.

Bone fixation was performed with two Kirschner wires. An end-to-end arterial anastomosis was performed using a vein graft from the foot's dorsum to connect the first dorsal intermetatarsal artery and the radial artery at the level of the anatomical snuffbox. The end-to-end venous anastomosis was performed in a comitant vein. The nerves were coapted primarily with 10-0 nylon. Flexor and extensor tenorrhaphies were performed with 4-0 polypropylene. Full thickness skin grafting was necessary to close the surgical site without tension ( Figs. 1 to 4 ).

## Postoperative Assessment

Five years after the procedure, the patient underwent a postoperative assessment performed by an occupational therapist and a hand surgeon. The now 9-year-old patient was completely adapted to his daily activities. The child regularly attends sports classes, has no pain in the operated foot, no running problems, can jump on one foot, and there is no complaint of frequent falls.

The Childhood Health Assessment Questionnaire (CHAQ) 4 was applied to evaluate how much the toe transfer impacted the daily activities. This questionnaire is answered by one of the patient's relatives, and contains eight domains (dressing, arising, eating, walking, hygiene, reach, grip, and activities) and a disability index that ranges from 0 to 3. The CHAQ also has a visual analog scale (VAS) for pain and another scale to assess the child's overall well-being, which ranges from 0 to 100. The result was 0 on the 8 domains of the CHAQ, the disability index, and both the VAS and the overall well-being scale. Furthermore, the patient's mother reported no difficulties in the daily activities, no disability, no pain, and an excellent overall well-being on the part of the child.

The Semmes-Weinstein monofilament and two-point discrimination tests (using the Touch-Test, North Coast Medical, Inc., Morgan Hill, CA, US) were performed. The patient felt the green Semmes-Weinstein monofilament (0.05 g) in the finger pulp (which is considered normal sensibility) and had a two-point discrimination value in the finger pulp of 2 mm.

The Jamar dynamometer (Sammons Preston, Bolingbrook, IL, US), calibrated in the second ring, and the Jamar Pinch Gauge (Sammons Preston) were used to assess the handgrip and pinch strength respectively ( Table 1 ).

**Table 1:** Strength assessment

	Right side	Left side
Tip pinch (kgf)	2.0	2.1
Tripod pinch (kgf)	2.3	4.0
Lateral pinch (kgf)	2.3	4.6
Handgrip strength (kgf)	5.6	18

Abbreviation: kgf, kilogram-force.

The Jebsen-Taylor Hand Function Test (JTHFT) 5 was applied ( Table 2 ), and it is used to assess hand function and quantify the time required to perform different tasks that mimic those performed in daily life.

**Table 2:** Time in seconds required to perform the Jebsen-Taylor Hand Function Test

	Dominant hand (left)	Non-dominant hand (right)
Writing	43.4	72.4
Turning over cards	6.8	5.1
Small objects	8.9	11.2
Simulated feeding	15.0	18.9
Stacking checker pieces	3.7	3.6
Large and light objects	4.2	3.9
Large and heavy objects	3.6	4.5

For purposes of comparison, we used the results presented by Rufino et al. 6 In the present study, the JTHFT was standardized for children aged between 6 and 10 years ( Table 3 ).

**Table 3:** Mean time in seconds and standard deviation of the Jebsen-Taylor Hand Function Test in normal children aged 6 to 10 years

	** Dominant hand**	** Non-dominant hand**
Writing	39.3 ± 15.4	79.4 ± 29.0
Turning over cards	5.3 ± 1.8	5.8 ± 1.7
Small objects	6.8 ± 1.2	7.6 ± 1.9
Simulated feeding	15.1 ± 5.1	21.2 ± 7.0
Stacking checker pieces	4.5 ± 1.0	5.0 ± 1.1
Large and light objects	3.9 ± 1.0	4.1 ± 0.9
Large and heavy objects	4.2 ± 1.1	5.4 ± 1.6

Comparing the data from ( Tables 2 and 3 ), it is possible to observe that the patient needs more time (11.2 seconds) to pick up small objects and less than the normal time to stack checker pieces (3.6 seconds) when using the operated hand. The other subtests were performed at normal times.

**Fig. 5:**
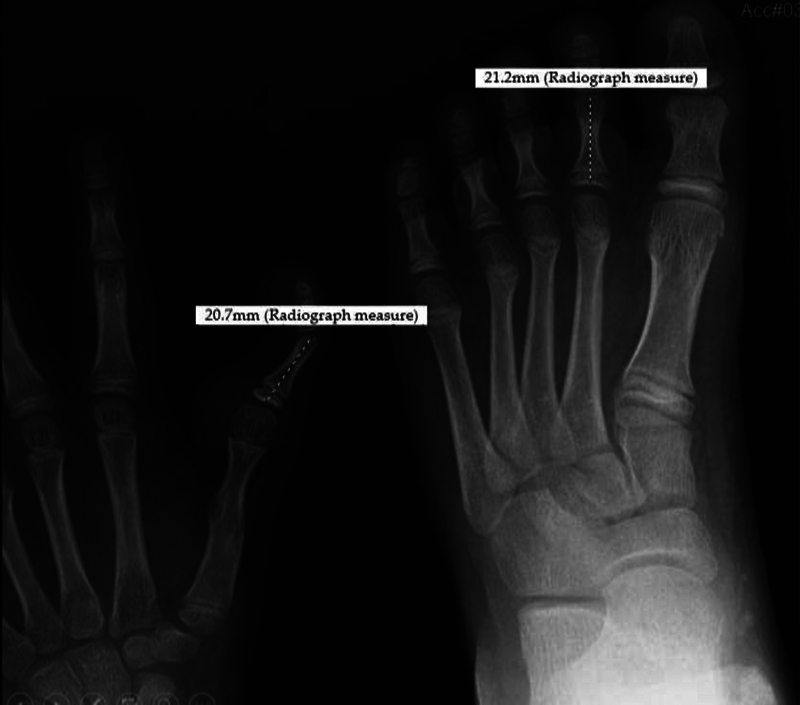
Radiograph of the right hand and foot, under the same magnification, with a comparative measure of the length of the proximal phalanx.

An X-ray was performed ( Fig. 5 ), which showed the proximal phalanx of the transplanted second toe with 20.7 mm and the proximal phalanx of the contralateral toe with 21.2 mm in length. It was also possible to observe the maintenance of the open physis.

## Discussion

J. R. Cobbett 7 performed the first reported successful microsurgical toe transfer in 1968. Since then, numerous reports and publications have been made demonstrating the effectiveness, safety, and satisfactory results of the procedure. 8

When compared to adults, the smaller size of the anatomical structures and the greater spasticity of the vessels in children are the main challenges of this procedure. 9

Although challenging, reconstructive surgeries in children, such as toe-to-hand transfer, are incredibly successful regarding the functional outcome. In the present case report, the patient showed excellent ability to develop daily activities, as demonstrated by the CHAQ and the JTHFT. The middle finger's flexion contracture, which was a consequence of the initial trauma extension to the palm, did not significantly impact the overall function ( Figs. 1 and 4 ).

In Fig. 4 , it is possible to see that the final esthetic outcome was not ideal. Transfer of a great toe would probably have resulted in a better esthetic outcome, with a less bulky finger pulp without a claw deformity. However, considering the significant morbidity of the donor site for the great toe transfer, the second toe is usually the best choice for children. 10

The children's ability to understand and comply with the tests is critical to obtain a reliable result. There is some scientific evidence that children older than 6 years of age are able to complete the standard tests of motor and sensory function, which are applied in cases of peripheral-nerve injuries. 11 Sensory recovery in children is usually better than in adults due to their greater ability to regenerate peripheral nerves and neuroplasticity. 8 In the present case, the recovery of a normal sensibility in the transplanted finger was achieved.

Few studies approach the growth potential of the toe transplanted to the hand. 12 In the case herein presented, the length of the proximal phalanx of the transplanted second toe is very similar to that of the contralateral toe, five years after the surgery. This radiographic analysis provided substantial evidence of the preservation of the epiphyseal plate and growth potential in pediatric toe-to-hand transfers.

Despite this procedure's technical difficulties, especially in children, our analysis of functional aspects, pain, sensibility, donor-site morbidity, and growth potential showed good results. These results provide supportive data for toe transfer in children with traumatic thumb loss.
