# Supplementary material for: Transfer of Second Toe for the Treatment of Traumatic Thumb Amputation in a Four-year-old Child: A Case Report
Source: Rev Bras Ortop (Sao Paulo). 2022 May 31;59(4):e632–6. doi: 10.1055/s-0042-1744518 (PMC11374401; doi:10.1055/s-0042-1744518)
Supplement: Related texts

# Research article: Transferência do segundo dedo do pé para tratamento de amputação traumática do polegar em uma criança de quatro anos: Relato de caso

**Keywords:** amputação traumática/cirurgia, criança, dedos do pé/ transplante, polegar

## Abstract

A transferência microcirúrgica do dedo do pé para a reconstrução do polegar é um procedimento desafiador, considerando-se a habilidade técnica necessária para realizá-la e a difícil avaliação pós-operatória dos aspectos estéticos e funcionais. Este é um relato do caso de uma criança de 3 anos que sofreu uma amputação traumática do polegar. A reconstrução do polegar foi realizada por meio de transferência microcirúrgica do dedo do pé, meses após a falha do reimplante. Uma avaliação subjetiva e outra objetiva dos resultados foram realizadas cinco anos após o procedimento. O resultado funcional foi avaliado por meio do Questionário de Avaliação da Saúde na Infância (Childhood Health Assessment Questionnaire, CHAQ, em inglês) e o Teste Funcional da Mão de Jebsen-Taylor (Jebsen-Taylor Hand Function Test, JTHFT, em inglês). Foram usados o dinamômetro Jamar (Sammons Preston, Bolingbrook, IL, EUA) e os instrumentos de medição Jamar Pinch Gauge (Sammons Preston) para avaliar as forças de preensão palmar e de pinças, respectivamente. Foram realizados os testes de monofilamento e de discriminação de dois pontos de Semmes-Weinstein. O paciente apresentou excelente resultado funcional, recuperação parcial da força, recuperação sensitiva completa, e mínima morbidade do sítio doador. Foi realizada também uma avaliação radiográfica, que demonstrou a preservação da placa epifisária e do potencial de crescimento no dedo do pé transplantado. Em todos os aspectos, os dados observados reforçam os benefícios e as indicações da transferência do dedo do pé para a reconstrução do polegar em crianças.

## Introdução

As amputações traumáticas do membro superior são responsáveis por impactos econômicos e sociais significativos. Existem poucos estudos epidemiológicos sobre as amputações dos dedos mas sabe-se que são mais frequentes em homens adultos jovens. 1

A amputação traumática do polegar é rara e dramática, e o reimplante sempre deve ser tentado. Mas, em caso de falhas, o procedimento de reconstrução do polegar, que inclui alongamento da parte remanescente, policização, reconstrução osteoplástica, e transferência do dedo do pé, deve ser considerado. 2

Entre todos os procedimentos disponíveis, a transferência do dedo do pé é o mais desafiador. No entanto, tem grande potencial de proporcionar um bom resultado final. 3

Considerando os diversos aspectos envolvidos na transferência do dedo do pé, principalmente em crianças, apresentamos um caso que destaca o longo acompanhamento clínico e a preservação do potencial de crescimento.

## Relato de Caso

Os pais do paciente assinaram o termo de consentimento livre e esclarecido. Este estudo foi aprovado pelo Comitê de Ética do hospital sob o número 4.075.812.

Em 2013, um menino de 3 anos sofreu uma amputação traumática do polegar direito por uma serra circular. O reimplante foi realizado, porém, devido a falha, o polegar teve que ser retirado na semana seguinte. Em 2014, foi realizada a transferência do 2º dedo do pé esquerdo para o polegar direito quando a criança tinha 4 anos.

Duas equipes cirúrgicas realizaram o procedimento. Durante a dissecção do segundo dedo do pé, foram identificados a primeira artéria intermetatarsal dorsal, que era a artéria dominante, assim como duas veias, dois nervos digitais, e os tendões flexores e extensores. Simultaneamente, a dissecção da mão incluiu a identificação da artéria radial, das veias receptoras, dos nervos digitais próprios e dos tendões extensor e flexor longo do polegar.

A fixação óssea foi realizada com dois fios de Kirschner. Uma anastomose arterial término-terminal foi realizada usando um enxerto venoso do dorso do pé para conectar a primeira artéria intermetatarsal dorsal e a artéria radial ao nível da tabaqueira anatômica. A anastomose venosa término-terminal foi realizada em veia comitante. Os nervos foram coaptados primariamente com fio de náilon 10-0. A tenorrafia flexora e extensora foi realizada com polipropileno 4-0. Foi necessário um enxerto de pele total para fechar o sítio cirúrgico sem tensão ( Figs. 1 a 4 ).

**Fig. 1:** Planejamento da incisão da mão.

**Fig. 2:** Planejamento da incisão do pé.

**Fig. 3:** Resultado pós-operatório imediato.

**Fig. 4:** Resultado pós-operatório estético e funcional tardio.

## Avaliação Pós-operatória

O paciente foi submetido a avaliação pós-operatória cinco anos depois do procedimento, feita por um terapeuta ocupacional e um cirurgião de mão. O paciente, agora com 9 anos, está totalmente adaptado às atividades diárias. A criança participa regularmente das aulas de educação física, e não apresenta dores no pé submetido à cirurgia ou problemas para correr e saltar em um único pé. O paciente também não apresentou queixas de quedas frequentes.

Foi aplicado o Questionário de Avaliação da Saúde na Infância (Childhood Health Assessment Questionnaire CHAQ, em inglês), 4 com o propósito de avaliar o quanto a transferência do dedo do pé interferiu nas atividades diárias. Este questionário é respondido por um familiar do paciente, e abrange oito domínios (vestir-se, levantar, comer, andar, higiene pessoal, alcance, pegada, e atividades) e um índice de incapacidade que varia de 0 a 3. O CHAQ também tem um escala visual analógica (EVA ) para dor e outra para avaliar o bem-estar geral da criança, que varia de 0 a 100. O resultado foi 0 para os oito domínios do CHAQ, no índice de incapacidade, na EVA e na escala de bem-estar geral. Além disso, a mãe do paciente não relatou dificuldades nas atividades diárias, nenhuma deficiência ou dor, além de um excelente bem-estar geral da criança.

Foram realizados os testes de monofilamento de Semmes-Weinstein e de discriminação de dois pontos (por meio do Touch-Test, North Coast Medical, Inc., Morgan Hill, CA, EUA). O paciente sentiu o monofilamento de Semmes-Weinstein verde (0,05 g) na polpa do dedo (sensibilidade considerada normal), e, no teste de discriminação de 2 pontos, apresentou um valor na polpa do dedo de 2 mm.

O dinamômetro Jamar (Sammons Preston, Bolingbrook, IL, EUA), calibrado no segundo anel, e o Jamar Pinch Gauge (Sammons Preston) foram utilizados para avaliar a força de preensão palmar e de pinças, respectivamente ( Tabela 1 ).

**Tabela 1:** Avaliação da força

	Lado direito	Lado esquerdo
Pinça digital (kgf)	2,0	2,1
Pinça Trípode (kgf)	2,3	4,0
Pinça lateral (kgf)	2,3	4,6
Força de preensão palmar (kgf)	5,6	18

Abreviatura: kgf, quilograma-força.

Foi aplicado o Teste Funcional da Mão de Jebsen-Taylor (Jebsen-Taylor Hand Function Test, JTHFT, em inglês) 5 ( Tabela 2 ), para avaliar a função da mão e quantificar o tempo necessário para executar diferentes tarefas que mimetizam as realizadas na vida diária.

**Tabela 2:** Tempo (em segundos) necessário para realizar o teste Funcional da Mão de Jebsen-Taylor

	Mão dominante (esquerda)	Mão não dominante (direita)
Escrever	43,4	72,4
Virar cartas	6,8	5,1
Objetos pequenos	8,9	11,2
Simular alimentação	15,0	18,9
Empilhamento de peças de damas	3,7	3,6
Objetos grandes e leves	4,2	3,9
Objetos grandes e pesados	3,6	4,5

Para fins comparativos, utilizamos os resultados apresentados por Rufino et al. 6 Neste estudo, o JTHFT foi padronizado para crianças entre 6 e 10 anos ( Tabela 3 ).

**Tabela 3:** Tempo médio (em segundos) e desvio padrão do Teste Funcional da Mão de Jebsen-Taylor em crianças normais de 6 a 10 anos de idade

	Mão dominante	Mão não dominante
Escrever	39,3 ± 15,4	79,4 ± 29,0
Virar cartas	5,3 ± 1,8	5,8 ± 1,7
Objetos pequenos	6,8 ± 1,2	7,6 ± 1,9
Simular alimentação	15,1 ± 5,1	21,2 ± 7,0
Empilhamento de peças de damas	4,5 ± 1,0	5,0 ± 1,1
Objetos grandes e leves	3,9 ± 1,0	4,1 ± 0,9
Objetos grandes e pesados	4,2 ± 1,1	5,4 ± 1,6

Comparando os dados das Tabelas 2 e 3 , é possível observar que, fazendo uso da mão operada, o paciente necessita de mais tempo (11,2 segundos) para pegar pequenos objetos e menos tempo do que o normal para empilhar as peças do jogo de damas (3,6 segundos). Os outros subtestes foram realizados em um tempo normal.

Foi realizada radiografia ( Fig. 5 ), que evidenciou a falange proximal do segundo dedo do pé transplantado com 20,7 mm, e a falange proximal do dedo do pé contralateral com 21,2 mm de comprimento. Também foi possível observar a manutenção da fise aberta.

**Fig. 5:** Radiografia da mão e pé direitos, sob a mesma ampliação, com medida comparativa do comprimento da falange proximal.

## Discussão

Em 1968, relatou-se a primeira transferência microcirúrgica do dedo do pé bem-sucedida realizada por J. R. Cobbett.7 Desde então, vários relatos e publicações foram feitos demonstrando a eficácia, a segurança e os resultados satisfatórios do procedimento. 8

Os principais desafios desse procedimento em crianças, em comparação com os realizados em adultos, são o menor tamanho das estruturas anatômicas e a maior vasoespasmo nas crianças. 9

Embora desafiadoras, as cirurgias reconstrutivas em crianças, como a transferência do dedo do pé para a mão, são incrivelmente bem-sucedidas em termos de resultado funcional. Neste relato de caso, o paciente apresentou excelente capacidade para realizar as atividades diárias, conforme demonstrado pelo CHAQ e pelo JTHFT. A contratura em flexão do dedo médio, decorrente da extensão do trauma inicial para a palma da mão, não afetou significativamente a função geral ( Figs. 1 e 4 ).

Na Fig. 4 , é possível perceber que o resultado estético final não foi o ideal. Uma transferência do hálux provavelmente teria dado um resultado estético melhor, proporcionando uma polpa do dedo menos volumosa, sem uma deformidade em garra. No entanto, considerando a morbidade significativa do sítio doador na transferência do hálux, o segundo dedo do pé geralmente é a melhor escolha quando se trata de crianças. 10

A capacidade das crianças de compreender e cumprir os testes é fundamental para que se obtenha um resultado confiável. Existem algumas evidências científicas de que as crianças maiores de 6 anos de idade são capazes de completar os testes padrão de função motora e sensorial, que são aplicados em casos de lesões dos nervos periféricos. 11 A recuperação sensorial em crianças geralmente é melhor do que em adultos, devido à maior capacidade de regeneração nervosa periférica e à neuroplasticidade. 8 Nesse caso, foi alcançada a recuperação da sensibilidade normal do dedo transplantado.

Poucos estudos abordam o potencial de crescimento do dedo do pé transplantado para a mão. 12 No caso apresentado, o comprimento da falange proximal do segundo dedo do pé transplantado é muito semelhante ao do dedo do pé contralateral cinco anos após a cirurgia. Esta análise radiográfica forneceu evidências substanciais da preservação da placa epifisária e do potencial de crescimento em transferências pediátricas do dedo do pé para a mão.

Apesar das dificuldades técnicas desse procedimento, principalmente em crianças, nossa análise dos aspectos funcionais, dor, sensibilidade, morbidade do sítio doador e potencial de crescimento mostraram bons resultados. Esses resultados fornecem dados favoráveis à transferência do dedo do pé em crianças com perda traumática do polegar.
